# Decreased mRNA expression levels of DNA methyltransferases type 1 and 3A in systemic lupus erythematosus

**DOI:** 10.1007/s00296-017-3711-8

**Published:** 2017-03-27

**Authors:** Mariusz J. Nawrocki, Dominik Majewski, Mariusz Puszczewicz, Paweł P. Jagodziński

**Affiliations:** 1 Department of Biochemistry and Molecular Biology, Poznań University of Medical Sciences, 6 Święcickiego St., 60-781 Poznań, Poland; 2 Department of Rheumatology and Internal Diseases, Poznań University of Medical Science, 135/147 28 Czerwca 1956 r. St., 61-545 Poznań, Poland

**Keywords:** SLE, Epigenetic alterations, DNA methylation

## Abstract

**Objectives:**

Systemic lupus erythematosus (SLE) is a chronic relapsing autoimmune disease characterized by the presence of autoantibodies directed against nuclear antigens and by chronic inflammation. Although the etiology of SLE remains unclear, the influence of environment factors, which is largely reflected by the epigenetic mechanisms, with DNA methylation changes in particular, is generally considered as main players in the pathogenesis of SLE. We studied DNA methyltransferases’ (DNMTs) type 1, 3A and 3B transcript levels in peripheral blood mononuclear cells from patients diagnosed with systemic lupus erythematosus and from the healthy control subjects. Furthermore, the association of DNMT1, DNMT3A, and DNMT3B mRNA levels with gender, age, and major clinical manifestations was analyzed.

**Methods:**

Peripheral blood mononuclear cells (PBMCs) were isolated from 32 SLE patients and 40 healthy controls. Reverse transcription and real-time quantitative polymerase chain reaction (RT-qPCR) analyses were used to determine DNMT1, DNMT3A, and DNMT3B mRNA expression levels.

**Results:**

Significantly lower DNMT1 (*p* = 0.015543) and DNMT3A (*p* = 0.003652) transcript levels in SLE patients were observed compared with healthy controls. Nevertheless, the DNMT3B mRNA expression levels were markedly lower compared with DNMT1 and DNMT3A, both in PBMCs from affected patients and those from control subjects. Furthermore, the DNMT1 transcript levels were positively correlated with SLE disease activity index (SLEDAI) (*r*_s_ = 0.4087, *p* = 0.020224), while the DNMT3A transcript levels were negatively correlated with patients age (*r*_s_ = −0.3765, *p* = 0.03369).

**Conclusions:**

Our analyses confirmed the importance of epigenetic alterations in SLE etiology. Moreover, our results suggest that the presence of some clinical manifestations, such as phototosensitivity and arthritis, might be associated with the dysregulation of DNA methyltransferases’ mRNA expression levels.

## Introduction

Systemic lupus erythematosus (SLE) is a chronic relapsing autoimmune disorder with a spectrum of clinical manifestations and outcomes [1]. SLE affects many tissues and organs (such as the skin, kidneys, joints, respiratory and nervous systems); nevertheless, etiology of this heterogeneous disorder remains incompletely described. Currently, it is established that SLE is the result of interactions between genetic, epigenetic, hormonal, and environmental factors [2–6]. SLE, such as many autoimmune diseases, occurs more frequently in women, and the reason for this female preponderance is suggested role of female sex hormones [7]. Recent advances in gene expression analyses, high-throughput single nucleotide polymorphism (SNP) genotyping, and association studies have identified genetic loci or genes that dictate immune abnormalities in autoimmune diseases [8, 9].

Nevertheless, multiple studies have demonstrated a pivotal role of epigenetic mechanisms of gene regulation in SLE etiology. In contrast to genetic alterations, an epigenetic change is defined as a heritable change in gene expression that does not involve a change in the DNA sequence [10]. Epigenetic mechanisms play an essential role in eukaryotic gene regulation by modifying chromatin structure, which in turn modulates gene expression. There are three processes that have been most frequently implicated in epigenetic control: histone modification, DNA methylation, and microRNA expression patterns [6]. Methylation of deoxycytosine (dC) bases in CG pairs, referred to as DNA methylation, is the most common epigenetic mechanisms of gene regulation. CG pairs are found ubiquitously throughout the genome. Among all the CpGs in the human genome, 6080% are generally methylated. Less than 10% of CpGs occur in CG-dense regions that are termed CpG islands [11]. However, their transcriptionally repressive activity is generally ascribed to promoter regions [12]. DNA methylation contributes to systemic lupus erythematosus predisposition and is generally considered as key player in the pathogenesis of SLE [13]. Twin studies confirmed the importance of DNA demethylation in human lupus [14].

**Fig. 1:**
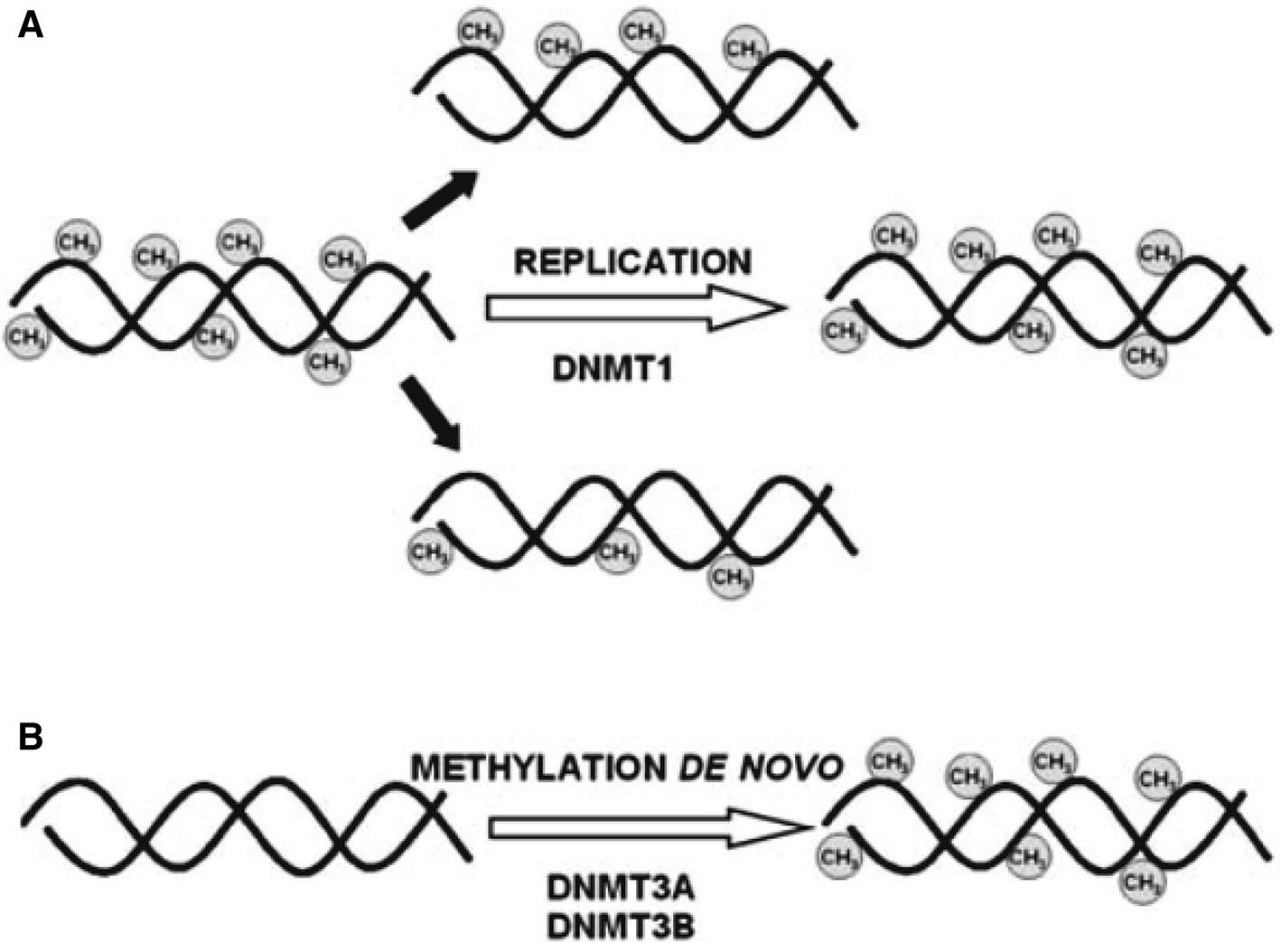
Maintenance (**a**) and de novo DNMTs (**b**) methylate DNA. DNMT1 binds methyl groups to the hemimethylated DNA during replication, whereas DNMT3A and DNMT3B can add methyl groups to CpG dinucleotides of unmethylated DNA. From Luczak and Jagodziński [23]

The addition of a methyl group to the fifth position of the cytosine pyrimidine ring, predominantly at CpG dinucleotides, is mediated by DNA methyltransferases (DNMTs). De novo methylation and maintenance methylation are two distinct processes that are required for the establishment and mitotic inheritance of tissue-specific methylation patterns (Fig. 1). Among group of the DNMTs, DNMT3A and DNMT3B catalyze de novo DNA methylation, and DNMT1 mediates the maintenance of DNA methylation [11]. DNMT1 is the maintenance DNA methylation enzyme, recognizing hemimethylated cytosines during the S phase of the cell cycle and copying these marks onto the nascent strand during replication [15, 16]. Studies with the embryonic stem cells (ES cells) with inactivated DNMT3A and DNMT3B genes by gene targeting, demonstrated blocks de novo methylation in ES cells and early embryos [17]. These results indicate that both DNMT3A and DNMT3B are required for genome-wide de novo methylation and are essential for mammalian development.

We studied DNMT1, DNMT3A, and DNMT3B transcript levels in peripheral blood mononuclear cells (PBMCs) from 32 patients diagnosed with SLE and from 40 individuals as a control group.

## Materials and methods

### Human subjects

Thirty-two peripheral blood samples were obtained from patients diagnosed with SLE at the Department of Rheumatology and Internal Diseases in Poznan, Poland (Table 1), and 40 samples from the control group (40 females; median age = 30, range 19–47, mean age ± SD = 30.8 ± 8.6) were collected at the Regional Blood Donation and Blood Treatment Center in Poznan, Poland. The healthy control subjects were selected from healthy blood donors without any rheumatologic conditions. At the time of blood donation, the median and mean age of the patients were 36 (range 19–74) and 37.9 ± 14.4 years, respectively. Based on the SLE disease activity index (SLEDAI), SLE patients were divided into 12 inactive (SLEDAI <6) and 20 active (SLEDAI ≥6) cases. Peripheral blood samples were collected from all the patients before they took any immunosuppressive drug, glucocorticoid or cytotoxic drugs to exclude the influence of drugs. The study procedures were approved by the Local Ethical Committee of Poznań University of Medical Sciences.

**Table 1:** Demographic and clinical characteristics of SLE patients

Parameter	SLE patients (*n* = 32)
Demographic data	Mean ± SD
Age (years)	37.9 ± 14.4
Disease duration (years)	9.9 ± 7.9
Gender (female/male)	30/2
SLEDAI	7.7 ± 6.1
Clinical manifestations	No (%)
Malar rash	10 (31.3)
Discoid rash	11 (34.4)
Phototosensitivity	18 (56.3)
Oral ulcers	14 (43.8)
Arthritis	9 (28.1)
Serositis	4 (12.5)
Renal disorders	16 (50)
Neurological disorders	12 (37.5)
Haematological disease	7 (21.9)
Immunological disorders	18 (56.3)
Anti-nuclear Ab	32 (100)
Anti-dsDNA Ab	13 (40.6)
Anti-Smith Ab	7 (21.9)
Anti-snRNP Ab	7 (21.9)
Anti-Ro Ab	4 (12.5)
Anti-La Ab	6 (18.8)
Anti-Scl-70 Ab	10 (31.3)

### Isolation of PBMCs

Human peripheral blood mononuclear cells were isolated using Ficoll density gradient centrifugation method. Ficoll is a neutral, high-mass, branched polysaccharide that simply dissolves in aqueous solutions [18]. Blood (3 mL) was gently layered on top of an equal volume of Ficoll® (Sigma) solution (without mixing the two layers) in a 15 mL conical tube. The samples were centrifuged at 600×*g* for 50 min at room temperature whit the brake in the off position. PBMCs are retained at the interface between the Ficoll and plasma layers. The layer containing the mononuclear cells was harvested in 1.5 mL microcentrifuge tubes. In the next step, cells were washed in phosphate buffered saline (PBS) supplemented with 2 mM EDTA. Finally, after centrifugation at 250×*g* for 10 min, cells were resuspended in TRIzol® Reagent (Invitrogen, USA) for downstream applications.

### Reverse transcription and real-time quantitative polymerase chain reaction (RT-qPCR) analyses

Total RNA from PBMCs was isolated according to the method published by Chomczyński and Sacchi [19]. RNA integrity was determined by denaturing agarose gel electrophoresis, and then, the RNA was quantified by measuring the optical density (OD) at 260 nm. RNA samples were treated with DNase I and reverse-transcribed into cDNA using Moloney Murine Leukemia Virus (M-MLV) Reverse Transcriptase from Invitrogen, Life Technologies (Grand Island, NY), according to the manufacturer’s protocol. RT-qPCR was conducted in a Light Cycler®480 Real-Time PCR System, Roche Diagnostics GmbH (Mannheim, Germany) with SYBR Green I as the detection dye. The target cDNA was quantified by the relative quantification method using a calibrator for patient or control PBMCs. For the calibrator, 1 μl of cDNA from all samples were mixed together, and successive dilutions were used to create a standard curve as described in the Relative Quantification Manual Roche Diagnostics GmbH (Mannheim, Germany). For relative quantification, we applied the E-Method algorithm. The DNMT1, DNMT3A, and DNMT3B transcript levels in each sample were standardized to the porphobilinogen deaminase (PBGD) transcript level as an internal control. The DNMT1, DNMT3A, and DNMT3B transcript levels in PBMCs from the patients with SLE and the controls were expressed as a multiplicity of the cDNA concentration in the calibrator. For the amplification reactions, 1 μL of total (20 μl) cDNA solution was added to 9 μL of 5× LightCycler® 480 SYBR Green I Master from Roche Diagnostics GmbH (Mannheim, Germany) with primers (Table 2). Agarose gel electrophoresis was applied to confirm the specificity of the amplified products.

**Table 2:** Primer sequences

Gene	Sequence (5′–3′)	Product size (bp)
DNMT1	F: GATGAGAAGAAGCACAGAAGT R: TCTTTGGGGGTCGTTTTGCG	149
DNMT3A	F: GGTGCTGTCTCTCTTTGATG R: ATGCTTCTGTGTGACGCTG	178
DNMT3B	F: GGAAGGAGTTTGGAATAGGG R: CCAGTGCCACCAGTTTGTC	183
PBGD	F: GCCAAGGACCAGGACATC R: TCAGGTACAGTTGCCCATC	160

### Statistical analysis

The normality of the observed patient data distribution was assessed by the Shapiro–Wilk test, followed by the Mann–Whitney *U* test to identify statistically significant differences between the compared mean values. Correlations were calculated using Spearman’s rank correlation coefficient (*r*_s_). *p* values <0.05 were considered to be statistically significant. Statistical analyses were performed using the STATISTICA 12 software.

## Results

**Fig. 2:**
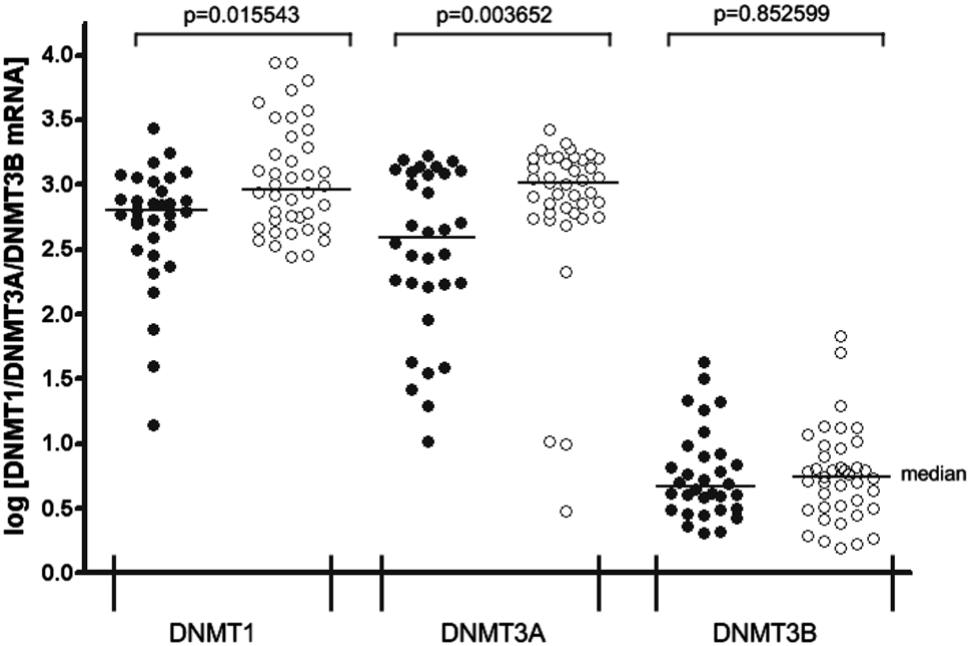
DNMT1, DNMT3A, and DNMT3B transcript levels in peripheral blood mononuclear cells (PBMCs) from patients with diagnosed SLE and control group. PBMCs from SLE patients (*n* = 32, *filled circles*) and controls (*n* = 40, *open circles*) were used for RNA isolation. Total RNA was reverse-transcribed, and cDNAs were investigated by RT-qPCR relative quantification analysis. The DNMT1, DNMT3A, and DNMT3B mRNA levels were corrected by PBGD level. The amounts of DNMT1, DNMT3A, and DNMT3B mRNA were expressed as the decimal logarithm of multiples of these cDNA copies in the calibrator

In the present study, employing RT-qPCR to compare the mRNA levels, DNMT1, DNMT3A, and DNMT3B transcripts in peripheral blood mononuclear cells from patients with SLE and healthy individuals were identified. Nevertheless, the DNMT3B transcript levels were markedly lower compared with DNMT1 and DNMT3A, both in PBMCs from affected patients (*p* < 0.00001 for DNMT1 and DNMT3A) and those from control subjects (*p* < 0.00001 for both enzymes also; Fig. 2). Significantly lower DNMT1 transcript expression (*p* = 0.015543) was observed in the 32 SLE patients than in the healthy controls. DNMT3A transcript levels also decreased significantly (*p* = 0.003652) in patients diagnosed with SLE compared with the controls. SLE patients and controls showed similar mRNA levels of DNMT3B (*p* = 0.852599).

**Fig. 3:**
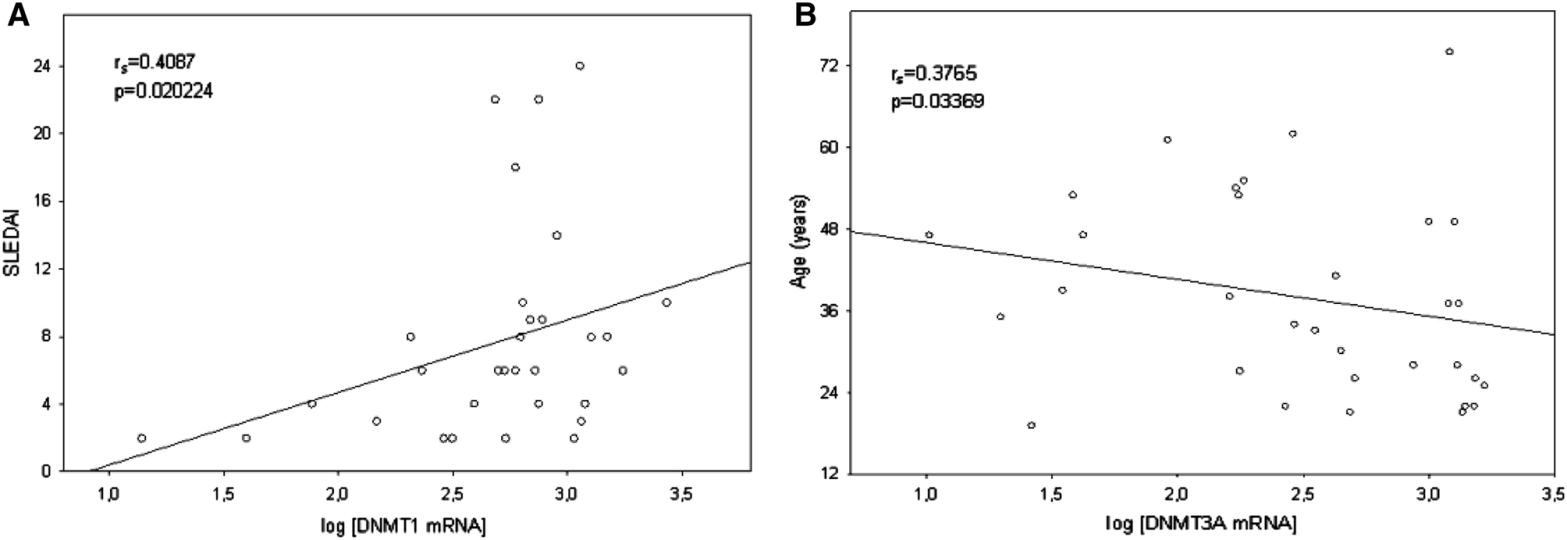
**a** Positive correlation between DNMT1 expression levels in PBMCs and SLEDAI in SLE patients (*n* = 32). **b** Negative correlation DNMT3A expression levels in PBMCs and SLE patients age (*n* = 32)

**Fig. 4:**
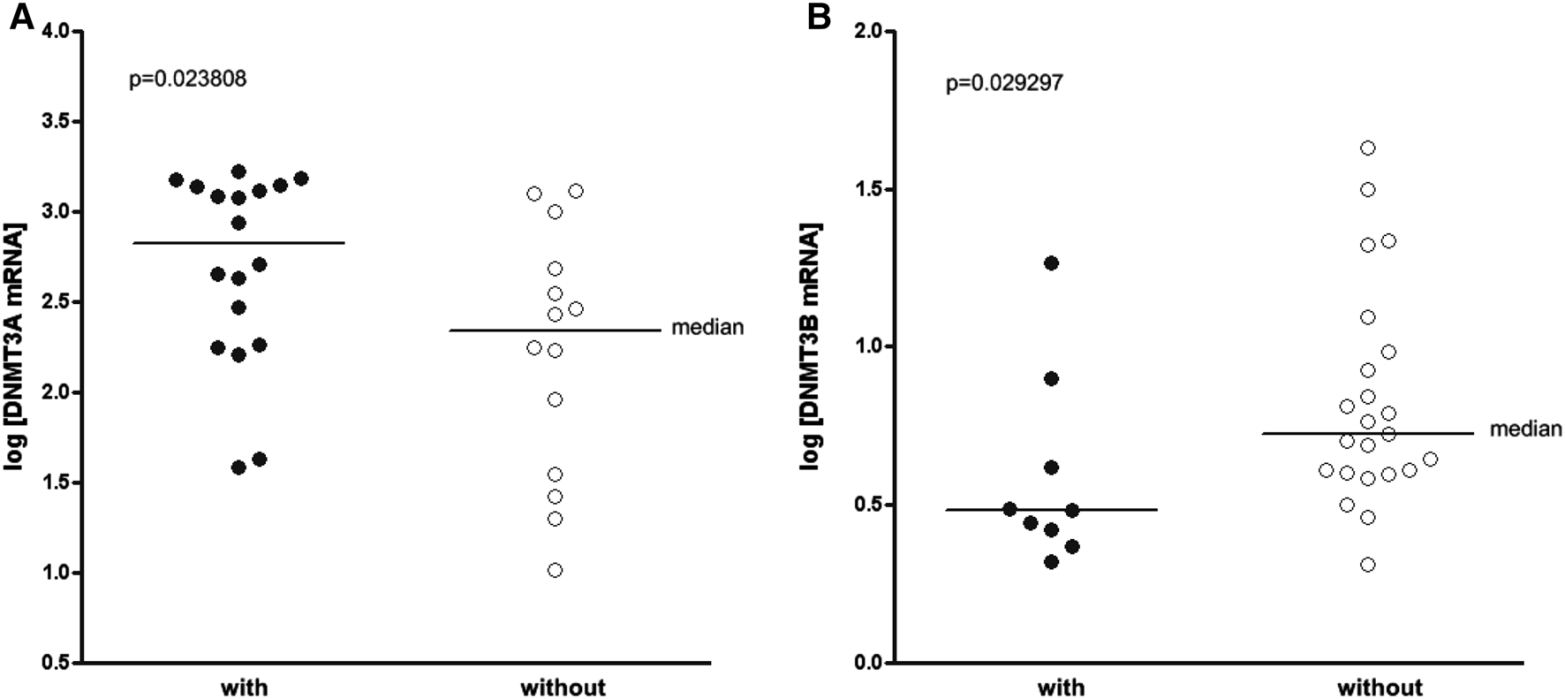
**a** DNMT3A mRNA levels in SLE patients with (*n* = 18, *filled circles*) and without (*n* = 14, *open circles*) photosensitivity. **b** DNMT3B mRNA levels in SLE patients with (*n* = 9, *filled circles*) and without (*n* = 23, *open circles*) arthritis

We also analyzed the relationships between DNMT1, DNMT3A, and DNMT3B mRNA expression levels and gender, age, and major clinical manifestations (listed in Table 1). Regarding the correlation of mRNA expression levels among SLE patients, DNMT1 transcript levels were positively correlated with SLE disease activity index (SLEDAI) (*r*_s_ = 0.4087, *p* = 0.020224; Fig. 3a). Interestingly, DNMT1 mRNA levels in active (SLEDAI ≥6) SLE patients were slightly higher compared with inactive (SLEDAI <6) SLE group; nevertheless, it was not statistically significant (*p* = 0.054007). Furthermore, the DNMT3A transcript levels were negatively correlated with patients age (*r*_s_ = −0.3765, *p* = 0.03369; Fig. 3b). This result suggests a lower mRNA expression levels of DNMT3A in older group of SLE patients (age >40); however, there was no statistically significant difference in the expression of DNMT3A mRNA among the different age groups (*p* = 0.076547). Substantial increase of DNMT3A mRNA amount in PBMCs (*p* = 0.023808) was observed in the group of patients with phototosensitivity (18 patients, 56.3%; Fig. 4a). Furthermore, individuals with arthritis (9 patients, 28.1%) were found to have significantly lower levels of DNMT3B mRNA levels (*p* = 0.029297) than patients without arthritis (Fig. 4b).

## Discussion

Genetic and epigenetic alterations unquestionably play a pivotal role in etiology of systemic lupus erythematosus (SLE). Studies of epigenetic aberrations in lupus patients have primarily focused on DNA methylation and various covalent histone modifications. DNA methylation is an epigenetic mark that is critical in determining chromatin accessibility and regulating gene expression. This epigenetic mechanism most frequently occurs at the fifth carbon of the pyrimidine ring in cytosine residues, primarily located in cytosine–guanosine dinucleotides (CpG dinucleotides). To this day have been described different patterns by which this epigenetic modification may affect chromatin accessibility. DNA methylation in the promoter regions is associated with gene silencing, thus linking DNA methylation to chromatin inaccessibility and gene suppression [20]. Interestingly, Hughes et al. [12] demonstrated that DNA methylation in gene promoter regions is not always a repressive epigenetic mark. Nevertheless, recent studies have also focused on the roles of DNA methylation in gene bodies and intergenic regions in enhancing gene expression [21, 22]. The mammalian DNA cytosine-5-methyltransferases’ (DNMTs) family comprises DNMT1, DNMT2, DNMT3A, DNMT3B, and DNMT3L; however, DNMT3L is primarily restricted to early embryogenesis, and does not play a major role [11, 16]. DNA methylation is established by the combined action of three DNMTs (DNMT2 functions to methylate RNA): DNMT1 is the maintenance methyltransferase and binds methyl groups to the hemimethylated DNA during replication, whereas DNMT3A and DNMT3B exhibit de novo methyltransferases’ activity and add methyl groups to CpG dinucleotides of unmethylated DNA to create new patterns of methylation [23, 24]. Thus, we analyzed the changes in transcript levels of DNMT1, DNMT3A, and DNMT3B in the whole PBMC population.

DNA methylation changes are closely related to the pathogenesis of SLE. Many studies have shown that abnormal DNA hypomethylation in some genes of CD4+ T cells can result in creation of autoreactive T cells and autoantibody generation. The most important methylation-sensitive T-cell genes include inter alia CD11a (encoded by gene ITGAL), perforin 1 (PRF1), CD70 (TNFSF7), CD40 ligand (TNFSF5), or killer-cell immunoglobulin-like receptors (KIR2DL4) [15, 24–27]. Other studies indicated that demethylation of inflammatory cytokine genes from interleukin family in CD4+ T cells might participate in the progression of SLE [28]. DNA methylation changes in regulatory regions of mentioned genes may be the result of action various factors, such as regulatory factor X 1 (RFX1) [29], overexpression of some microRNA [30–32], or long non-coding RNAs (lncRNAs) [33], which through interacting with DNMTs affects DNA methylation at distal target promoters. Other research found that Ras–mitogen-activated protein kinase (MAPK) and extracellular receptor associated kinase (ERK) pathway were involved in DNA hypomethylation by down-regulating DNMT activity in SLE [34, 35]. To confirm the importance of DNA hypomethylation in the progression of SLE, in recent years, few laboratories performed high-throughput genome-scale DNA methylation analysis and confirmed that the methylation level of many genes contributed to the development of diversified manifestations in SLE [14, 36–38]. Furthermore, by genome-scale DNA methylation analysis, Chung et al. found hypomethylation of CpG sites within genes from different pathways, which were involved in the production of autoantibodies to nuclear antigens in SLE [39].

In our study, using RT-qPCR analysis, we found decreased expression of DNMT1 and DNMT3A mRNA levels in SLE patients compared with the healthy individuals. As a consequence, and based on our results, we made the assumption that PBMCs from patients with SLE may be characterized by a global DNA hypomethylation. Other investigators demonstrated that both entire pool of PBMCs [40] and isolated T cells [41–43] from patients with SLE had a global DNA hypomethylation. Balada et al. [42] also demonstrated that CD4+ T cells from SLE patients had a low DNA methylcytosine content. Nevertheless, in contrast to our results, authors have shown similar mRNA levels of DNMT1, DNMT3A, and DMT3B in patients and control groups. It should be pointed out clearly that these authors used only purified CD4+ T cells from the entire pool of mononuclear cells. Overall, all enzymes reflected a wide range of variability among individuals, especially DNMT1 and DNMT3A. However, authors in other studies found that T cells [35] or PBMCs [44] from patients with lupus have exhibited a reduced DNMT1 mRNA levels. Some authors suggest that a defect in DNMT1 may be the primary reason of abnormal DNA methylation in CD4+ T cells from lupus patients [25]. Completely different results were achieved by Liu laboratory, and authors demonstrated significantly increased DNMT1 mRNA expression in SLE patients compared with the controls [40]. Other investigators [43, 45] described significantly higher mRNA levels of other enzymes (MBD1, MBD3, and MBD4), involved in the DNA methylation process, in patients with lupus. Therefore, these investigations suggest that the global DNA methylation may be dependent on multiple factors, such as DNMT mRNA levels, DNMT activity, DNMT protein levels, and transcript levels of other enzymes involved in the DNA methylation process. Nevertheless, our results confirmed the importance of epigenetic alterations in SLE etiology. Decreased expression of the DNMT1 and DNMT3A might be associated with generation of autoreactive T cells and autoantibody production. According to our results, in Balada studies [42], authors observed higher transcript levels of DNMT1 (moreover also for the DNMT3A) in active SLE patients. In the present study, we demonstrated markedly lower transcript levels of DNMT3B in PBMCs from SLE patients and those from control group—other studies also confirmed these observations [32, 42]. These results suggest that DNMT3B seems to be transcribed at low levels in PBMCs. Therefore, we may postulate that DNMT3A is the main enzyme which exhibits de novo methyltransferases’ activity in PBMCs both from affected patients and those from control subjects to create new patterns of methylation. Subsequent studies of Balada laboratory [27] also demonstrated that SLE patients had significantly less CD4+ T-cell DNA deoxymethylocytosine content than controls. As shown in this manuscript, ITGAL and PRF1 mRNA levels were directly and strongly correlated with the transcript levels of three DNMTs playing the lead role in DNA methylation.

In the present study, we also found positive correlation between DNMT1 transcript levels and SLE disease activity index (SLEDAI). Interestingly, our results suggest slightly higher mRNA levels of DNMT1 in group of patients with active (SLEDAI ≥6) SLE. The emerging discrepancy may be associated, as suggested by some [44] investigators, with corticosteroid treatment. Ogasawara et al. have reported that the levels of DNMT1 mRNA by peripheral blood mononuclear cells from patients with SLE were increased after corticosteroid treatment [44]. Moreover, previously published data support the hypothesis that cells respond to pharmacological or dietetical inhibition of methylation by induction of methyltransferase activity [46, 47]. Some authors [48] suggested that the DNMT activity is itself regulated, in part, by DNA methylation status, which represents a feedback mechanism. Investigators have found a regulatory region in murine *dnmt1* that seems to act as a sensor of the DNA methylation capacity of the cell. Furthermore, we observed an inverse correlation between the DNMT3A transcript levels and patients age. Whereas Zhang et al. [49] also found that the levels of DNMT1 and DNMT3A decreased with aging and that this change correlated with the hypomethylation of sequences flanking the ITGAL promoter. Other studies have shown that in DNA from aged subjects (65–90 years) compared with young subjects (20–35 years) is significantly decreased global level of methylation suggesting that age-associated hypomethylation of the DNA may be the cause of its increased immunogenicity. This change may thus be another mechanism that may contribute to the increase in age-associated chronic inflammation and autoimmunity. Moreover, delivery of DNA from aged compared with young subjects into PBMCs stimulated a significantly greater secretion of an inflammatory cytokine (IFN-α) and costimulatory markers CD80 and CD86 [50]. Therefore, we may postulate that a decrease of DNMTs expression during adulthood may be the pathway to development of autoimmunity with aging. Our results suggest also that the presence of some clinical manifestations, such as phototosensitivity and arthritis, might be associated with the dysregulation of DNA methyltransferases mRNA expression levels. As reviewed by Medlin et al. [51] in pooled analysis of SLE patients, most cutaneous manifestations (including phototosensitivity) were less prevalent in the late-onset SLE patients. Studies with Xeroderma pigmentosum (genetic disease characterized by hypersensitivity to UV exposure, associated with numerous skin abnormalities) skin cells indicate enormous importance of CpG methylation on frequency of targeted mutagenesis (TM) [52]. The distribution of TM events within the amplicon population was strongly biased toward demethylated sequences. Increase of TM frequency was observed when the DNMT1 gene was knocked down using specific siRNA. In addition, no induction of targeted mutagenesis after 5-aza-dC treatment was observed in the cells. Obtained by us results indicate slightly higher DNMT3A transcript levels, which may be associated with cells respond to pharmacological or dietetical inhibition of DNMTs activity. Less significance seems to have changes in the DNMT3B mRNA level changes associated with arthritis, because according to our results, this enzyme plays only a supporting role in methylation in PBMCs.

In our study, we analyzed the changes in transcript levels of DNMT1, DNMT3A, and DNMT3B in the whole PBMCs population. Note, mRNA levels in each group of immune cells may differ from the overall results obtained for PBMCs. For example, our results better reflect epigenetic changes in CD4 T cells (represent range of 25–60% of PBMC) than in B cells (up to 15% of PBMC). In humans, the frequencies of these populations vary across individuals, and also cell composition might be different in healthy controls and patients. The main limitation of our study is the lack of correlation between protein and mRNA levels. Keeping in mind the small amount of samples, we decided to investigate only DNMT1, DNMT3A, and DNMT3B mRNA levels.

## Conclusion

Our analyses confirmed the importance of epigenetic alterations in SLE etiology. Decreased expression of the two DNA cytosine-5-methyltransferases (DNMT1 and DNMT3A) might be associated with generation of autoreactive T cells and autoantibody production. We have shown that DNMT3A is the main enzyme which exhibits de novo methyltransferases activity in PBMCs both from affected patients and those from control subjects. In addition, our result can indicate the way by which it is achieved DNA hypomethylation in some specific genes in SLE pathogenesis.
